# Z-360, a novel therapeutic agent for pancreatic cancer, prevents up-regulation of ephrin B1 gene expression and phosphorylation of NR2B via suppression of interleukin-1 β production in a cancer-induced pain model in mice

**DOI:** 10.1186/1744-8069-6-72

**Published:** 2010-10-28

**Authors:** Yuki Orikawa, Hiroki Kato, Koichi Seto, Nobuyoshi Kobayashi, Koji Yoshinaga, Hiroki Hamano, Yuko Hori, Tim Meyer, Mineo Takei

**Affiliations:** 1 Central Research Laboratories, Zeria Pharmaceutical Co., Ltd., 2512-1 Numagami, Oshikiri, Kumagaya-shi, Saitama, Japan; 2 Department of Clinical Research, Zeria Pharmaceutical Co., Ltd., 10-11, Nihonbashi, Kobuna-cho, Chuo-ku, Tokyo, Japan; 3 UCL Cancer Institute, University College London, 72 Huntley Street, London, WC1E 6BT, UK

## Abstract

**Background:**

Z-360 is an orally active cholecystokinin-2 (CCK2)/gastrin receptor antagonist currently under development as a therapeutic drug for pancreatic cancer. It was previously reported that Z-360 treatment in combination with gemcitabine prolonged the survival period in a lethal pancreatic cancer xenograft model in mice. In a phase Ib/IIa clinical study, Z-360 treatment displayed a trend of reduced pain in patients with advanced pancreatic cancer in combination with gemcitabine including analgesics such as opioids. Here, we investigated the mechanism of analgesic action of Z-360 in a severe cancer-induced pain model in mice, which is considered to be opioid-resistant, by examining ephrin B1 gene expression, *N*-methyl-D-aspartate receptor NR2B subunit phosphorylation, and interleukin-1β (IL-1β) production.

**Results:**

In a mouse model of cancer-induced pain, ephrin B1 gene expression in dorsal root ganglia (DRGs) and the phosphorylation of NR2B in the spinal cord were induced. Z-360 treatment inhibited both ephrin B1 gene expression and the phosphorylation of NR2B. In addition, IL-1β production increased in the cancer-inoculated hind paw of mice, but could be suppressed by treatment with Z-360. Moreover, we observed that the CCK1 receptor antagonist devazepide similarly suppressed up-regulation of ephrin B1 gene expression and IL-1β production, and that the intraperitoneal injection of sulfated CCK-8 induced the production of IL-1β in the cancer-inoculated region.

**Conclusions:**

We have identified a novel pain cascade, in which IL-1β production in cancer-inoculated regions induces ephrin B1 gene expression in DRGs and then ephrin B1 enhances the tyrosine phosphorylation of NR2B via Eph B receptor in the spinal cord. Notably, Z-360 relieves cancer-induced pain by preventing this pain cascade through the suppression of IL-1β production, likely via the blockade of CCK1 receptor. The pre-clinical results presented here support the analgesic action of Z-360 in pancreatic cancer patients with severe, opioid-resistant pain. Pre-clinical and clinical results have demonstrated that Z-360 combined with gemcitabine represents a promising pancreatic cancer therapy approach with characteristic analgesic effects in addition to the prolongation of survival.

## Background

Patients with cancer develop diverse symptoms, among which pain is a major factor that can drastically reduce quality of life [1-3]. Pancreatic cancer, in particular, is often accompanied by severe pain, with 90% of patients reporting significant pain [4]. Cancer-induced pain is typically relieved with opioids, such as morphine, non-opioid analgesics, such as acetaminophen, and nonsteroidal anti-inflammatory drugs; however, these drugs are not uniformly effective and undesirable side effects often limit their use [5]. For these reasons, it is therefore desirable to develop novel medical agents with increased efficacy and safety for combating cancer-induced pain.

Z-360, calcium bis [(*R*)-(-)-3-[3-{5-cyclohexyl-1-(3,3-dimethyl-2-oxo-butyl)-2-oxo-2,3,4,5-tetrahydro-1*H*-benzo[*b*][1,4]diazepin-3-yl}ureido]benzoate], is an orally active 1,5-benzodiazepine derivative cholecystokinin-2 (CCK2)/gastrin receptor antagonist that is currently under development for the treatment of pancreatic cancer [6]. In pre-clinical studies, Z-360 inhibited pancreatic cancer growth and prolonged survival in combination with gemcitabine, a first line drug for the treatment of pancreatic cancer, in mouse pancreatic cancer xenograft models [7-9]. Z-360 was safe, well tolerated, and showed a trend towards reduced pain in phase Ib/IIa clinical study patients with advanced pancreatic cancer when used in combination with gemcitabine and analgesics, such as opioids [6].

In a previous pre-clinical study, we evaluated the analgesic effect of Z-360 using a mouse model of cancer-induced pain that was generated by the orthotopic transplantation of B16/BL6 melanoma cells into the plantar region of the right hind paw of C57BL/6 mice [10]. This model induced licking behaviour (an indicator of spontaneous pain), and mechanical allodynia and hyperalgesia in the periphery of the melanoma mass were observed from approximately day 10 after transplantation [11,12], with intensities that were a notable feature of this model. The mechanical allodynia and hyperalgesia occurring at a late phase in this model were considered to be resistant to existing analgesics, including opioids and adjuvant analgesics [11-13], which is similar to the resistance displayed by moderate-to-severe pain of cancer patients. Therefore, the effects of Z-360 on cancer-induced pain were examined in this model, and it was found that it displayed an analgesic effect that was attributed to the blockade of CCK1 receptor [10]. Moreover, we observed that the combination of Z-360 and morphine were more effective than when each were administered alone. For these reasons, the analgesic effect of Z-360 on cancer-induced pain model in mice is considered to be associated with the observed clinical benefit of Z-360 for pain relief against opioid-resistant pancreatic cancer pain [6]. Despite the evidence from these studies, the detailed analgesic mechanism of Z-360 is unclear.

Recent studies suggest that ephrin B-Eph B receptor signalling in dorsal root ganglia (DRGs) contributes to activation of the *N*-methyl-D-aspartate (NMDA) receptor [14,15] and plays a critical role in neuropathic pain [16,17] and inflammatory hyperalgesia [14]. We speculate that ephrin B-Eph B receptor signalling in DRGs is also involved in cancer-induced pain, as this type of pain is both neuropathic and inflammatory [12]. Interleukin-1β (IL-1β) is a major inflammatory cytokine that has diverse effects on the immune system and is considered to be relevant to cancer development, owing to high levels of serum IL-1β [18,19] and elevated gene expression [20,21] in cancer patients. Recently, it was reported that IL-1β gene promoter single nucleotide polymorphisms (IL-1β secretory phenotype) are associated with an increased risk for pancreatic cancer [19] and that IL-1β participates in opioid-resistant pain, such as bone cancer and neuropathic pain [22-27]. Zhang et al. also reported that spinal IL-1β facilitated bone cancer pain [24], and the intraneural administration of IL-1β into rat sciatic nerves induced signs of neuropathic pain [22]. Conversely, the administration of IL-1β inhibitors exhibited anti-hypernociceptive action in different models of neuropathic pain [26,27]. Based on these results, IL-1β is considered to be a critical factor in the development of opioid-resistant pain, such as cancer-induced pain. Based on the results of these studies, we therefore hypothesised that ephrin B-Eph B receptor signalling and IL-1β play a role in cancer-induced pain.

In this study, we attempted to determine the mechanism by which Z-360 mediates analgesic effects using a mouse model of cancer-induced pain and examining ephrin B-Eph B receptor signalling in DRGs, NMDA receptor NR2B subunit phosphorylation, and IL-1β production.

## Results

### Ephrin B1 gene expression in DRGs was induced in a cancer-induced pain model in mice

**Figure 1:**
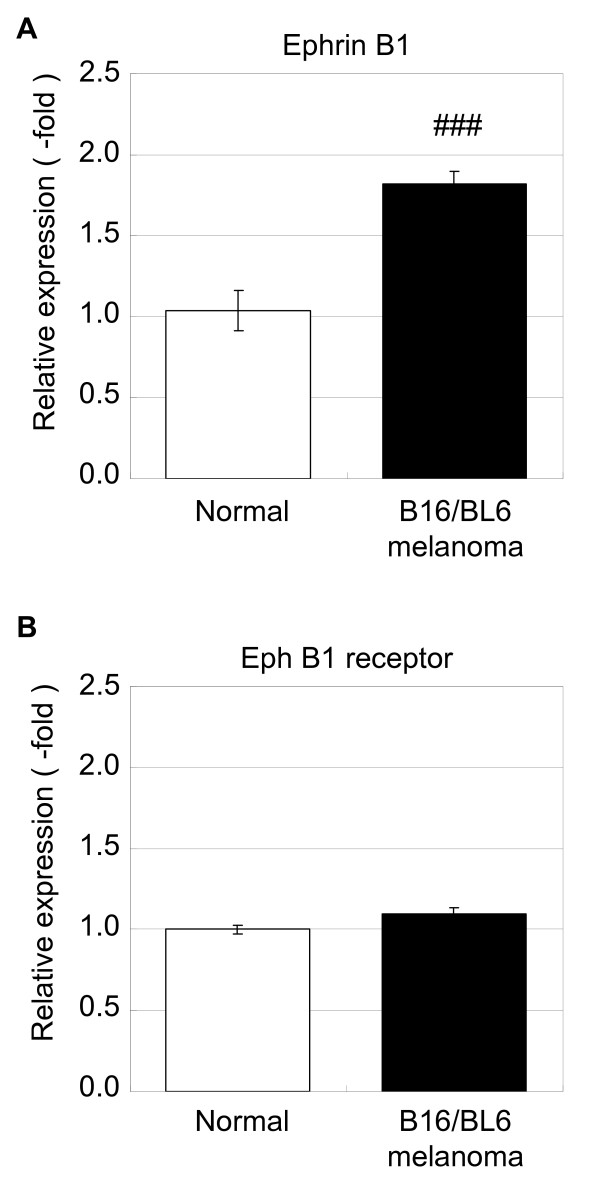
**Ephrin B1 and Eph B1 receptor gene expression in DRGs from cancer-induced pain mouse model**. B16/BL6 melanoma cells were injected into the plantar region of the right hind paw of C57BL/6 mice. L4-5 dorsal root ganglion (DRG) samples were taken on day 15 after transplantation. Gene expression analyses for ephrin B1 (A) and the Eph B1 receptor (B) were performed by quantitative real-time RT-PCR. Data represent fold changes versus the mean for normal mice. Columns represent the mean ± S.E. (n = 6). ###, p < 0.001, normal versus B16/BL6 melanoma (t-test).

In order to identify the factors that are associated with cancer-induced pain, we first examined the expression of several genes involved in the ephrin signalling pathway, including ephrin A1, ephrin A2, ephrin B1, ephrin B2, and the Eph A1 and Eph B1 receptors, in the DRGs of six-week old C57BL/6 mice injected with B16/BL6 melanoma cells into the plantar region of the right hind paw. As determined by quantitative real-time RT-PCR, the gene expression of ephrin B1 in lumbar (L) 4-5 DRGs was induced 1.8 fold in cancer-induced pain mice compared to normal control mice on day 15 after melanoma transplantation (p < 0.001) (Figure 1A); however, no change was observed for the Eph B1 receptor (Figure 1B). The expression of other examined genes (ephrin A1, A2, B2 and Eph A1 receptor) were also not induced (data not shown).

### Effect of Z-360 on ephrin B1 gene expression in a cancer-induced pain model in mice

**Figure 2:**
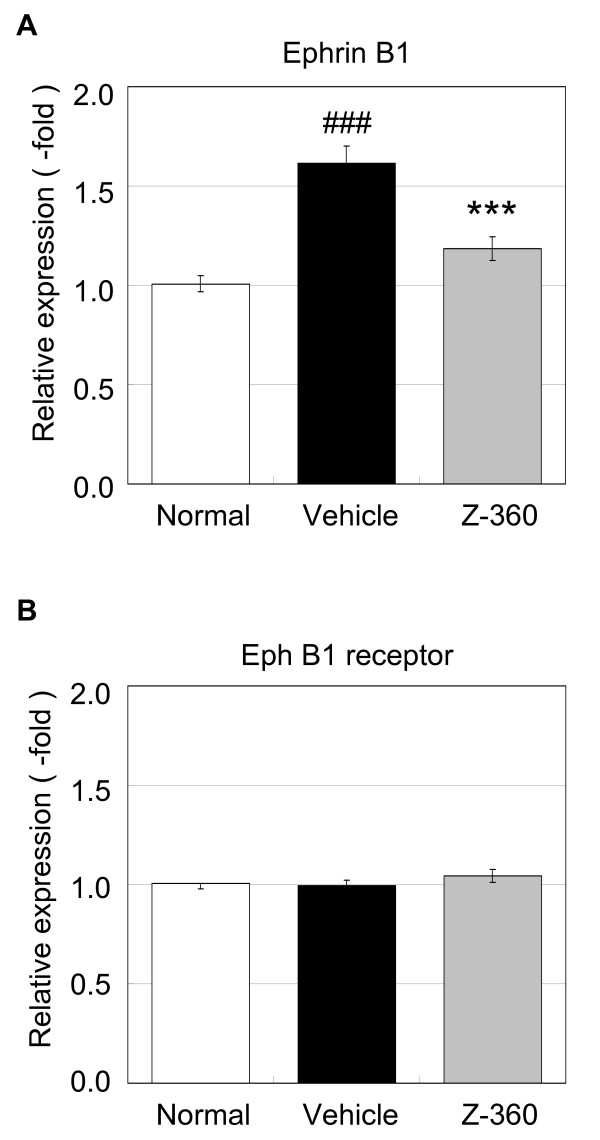
**Effects of Z-360 on ephrin B1 and Eph B1 receptor gene expression in DRGs**. B16/BL6 melanoma cells were injected into the plantar region of the right hind paw of C57BL/6 mice, and Z-360 (100 mg/kg) was then administered orally once a day from day 7 after transplantation. A final oral administration of Z-360 was given to mice 60 min prior to the collection of L4-5 DRG samples on day 14 after transplantation. Gene expression analysis for ephrin B1 (A) and the Eph B1 receptor (B) were performed by quantitative real-time RT-PCR. Data represent -fold changes versus the mean for normal mice. Columns represent the mean ± S.E. (n = 12). ###, p < 0.001, versus normal (t-test). ***, p < 0.001, versus vehicle (t-test).

After demonstrating that ephrin B1gene expression was up-regulated in DRGs of mice injected with B16/BL6 melanoma cells, the effect of Z-360 treatment was examined. Z-360 (100 mg/kg) was administered orally once a day beginning on day 7 to day 14 after transplantation, because the analgesic effect of Z-360 in cancer-induced pain was confirmed most strongly by repeated administration of a dose of 100 mg/kg [10]. On day 14 after transplantation, the expression of the ephrin B1 and Eph B1 receptor genes in L4-5 DRGs were examined (Figure 2A, B). Repeated administration of Z-360 suppressed the gene expression of ephrin B1 by 70.7% compared with the vehicle-treated group in cancer-induced pain mice (p < 0.001) (Figure 2A). The gene expression of the Eph B1 receptor was unchanged in all of the test groups (Figure 2B).

### Effect of Z-360 on NR2B phosphorylation in cancer-induced pain model in mice

**Figure 3:**
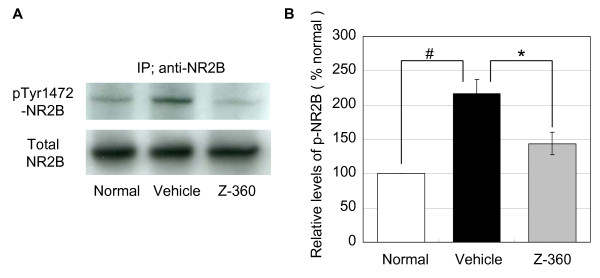
**Effect of Z-360 on spinal cord NR2B phosphorylation in the mouse model of cancer-induced pain**. A. NR2B was immunoprecipitated from lysates of the spinal cord of cancer-induced pain model in mice. The immunoprecipitates were separated on 10% SDS-PAGE gels and then transferred onto PVDF membranes with a Trans-Blot Cell System. The membranes were first probed with an antibody against pTyr1472-NR2B, stripped, and then reprobed with an antibody against NR2B. The panels show representative immunoblots of normal control, vehicle control, and Z-360 (100 mg/kg) treatment performed with antibodies against pTyr1472-NR2B (top) and NR2B (bottom). B. Histograms showing the relative tyrosine phosphorylation of NR2B normalized to normal controls. Adobe Photoshop Elements 5.0 software was used for densitometric quantification. Values represent the mean ± S.E. (n = 3). #, p < 0.05, normal versus vehicle (t-test); *, p < 0.05, vehicle versus Z-360 (t-test).

Several tyrosine residues in the C-terminal cytoplasmic region of NR2B have been shown to be phosphorylated by Src family tyrosine kinases *in vitro *[28], with Tyr-1472 being the major phosphorylation site. As it has been reported that ephrin B2 induces phosphorylation of Tyr-1472 residues [14], we next investigated whether Tyr-1472 phosphorylation of NR2B was enhanced in the spinal cord of the mouse model of cancer-induced pain. The level of phosphorylated Tyr-1472 NR2B in the vehicle control in cancer-induced pain mice was 210% higher than the normal control samples (p < 0.05) (Figure 3A, B); however, the repeated administration of Z-360 (100 mg/kg) once a day from day 7 to 14 after transplantation suppressed phosphorylation by 62.5% compared with the vehicle control (p < 0.05) (Figure 3A, B).

### IL-1β levels increased in the cancer-inoculated region of the right hind paw in the mouse model of cancer-induced pain and was suppressed by Z-360 treatment

**Figure 4:**
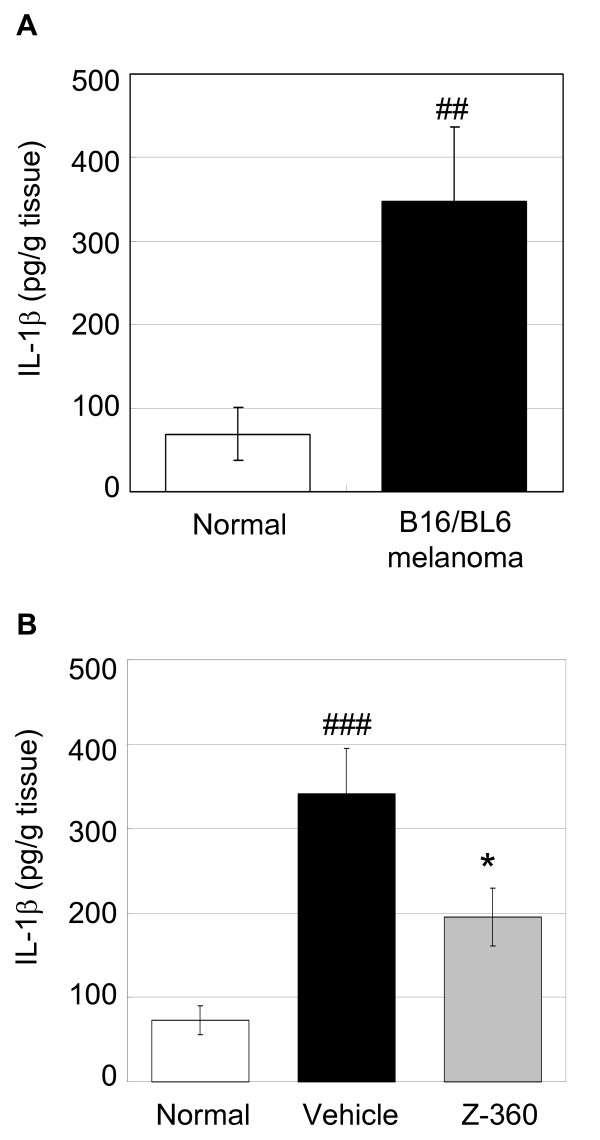
**IL-1β production in the cancer-inoculated region and the effect by Z-360 treatment**. A. B16/BL6 cells were injected into the plantar region of the right hind paw of C57BL/6 mice. On day 14 after transplantation, right hind paw samples were collected, homogenized, and subjected to an ELISA for the measurement of IL-1β. The results are reported as the mean ± S.E. (n = 12). ##, p < 0.01, normal versus B16/BL6 melanoma (Wilcoxon test). B. Z-360 (100 mg/kg) was administered orally once a day from day 7 to 14 after transplantation. A final dose of Z-360 was orally administered, and after 60 min, the levels of IL-1β were measured. The results are reported as the mean ± S.E. (n = 23-24). ###, p < 0.001, normal versus vehicle (Wilcoxon test); *, p < 0.05, vehicle versus Z-360 (Wilcoxon test).

To investigate the effects of increased gene expression of ephrin B1 in the mouse model of cancer-induced pain, we examined the change in the inflammatory mediator IL-1β in the cancer-inoculated region of the right hind paw by double sandwich enzyme-linked immunosorbent assay (ELISA). We found that IL-1β levels were significantly increased in these regions of cancer-induced pain mice compared with normal mice (p < 0.01) (Figure 4A). However, the administration of Z-360 (100 mg/kg) once a day from day 7 to 14 after transplantation significantly reduced the levels of IL-1β protein in the cancer-inoculated region by 54.3% compared to the vehicle-treated group in cancer-induced pain mice (p <0.05) (Figure 4B). This data suggested that Z-360 suppressed the production of IL-1β. In addition, the effect of Z-360 on tumour size was assessed at day 14 after transplantation. The tumour size of the Z-360-treated group was similar to that of vehicle-treated group (data not shown), indicating that Z-360 had no effect on tumour size in this cancer-induced pain model in mice.

### Effect of IL-1β injection into the right hind paw on the gene expression of ephrin B1

**Figure 5:**
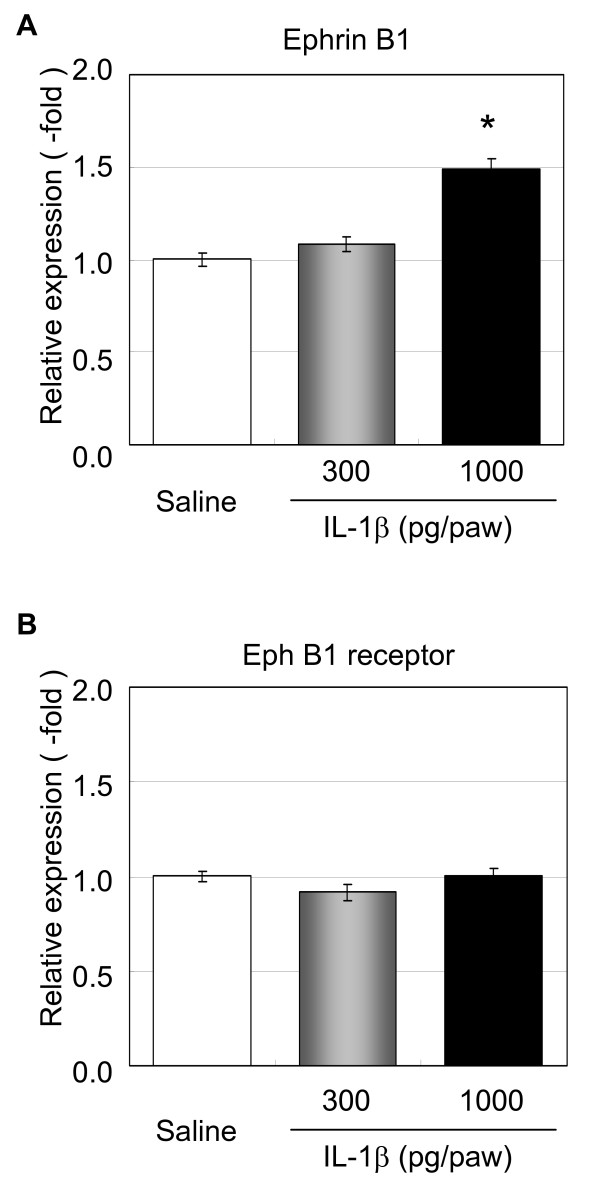
**Effects of IL-1β injection on ephrin B1 and Eph B1 receptor gene expression in DRGs**. Recombinant IL-1β (300 or 1000 pg/paw) was injected into the plantar region of the right hind paw of C57BL/6 mice, and L4-5 DRG samples were then collected after 60 min. Gene expression analysis for ephrin B1 (A) and the Eph B1 receptor (B) were performed by quantitative real-time RT-PCR. Data represent fold changes versus the mean for saline-injected control mice. Columns represent the mean ± S.E. (n = 4). *, p < 0.05, versus saline (non-parametric Dunnett's test).

To confirm whether IL-1β induced the gene expression of ephrin B1 in L4-5 DRGs, we injected IL-1β (300 and 1000 pg/paw) directly into the plantar region of the right hind paw of eight-week old C57BL/6 mice, and examined the expression of the ephrin B1 and Eph B1 receptor genes in L4-5 DRGs after 60 min. It was revealed that IL-1β injection (1000 pg/paw) into the plantar region of the right hind paw significantly induced the gene expression of ephrin B1 in L4-5 DRGs (p < 0.05), but not the Eph B1 receptor (Figure 5A, B). The injection IL-1β at 300 pg/paw had no effect on either ephrin B1 or Eph B1 receptor gene expression.

### Effects of devazepide on ephrin B1 gene expression and IL-1β production

**Figure 6:**
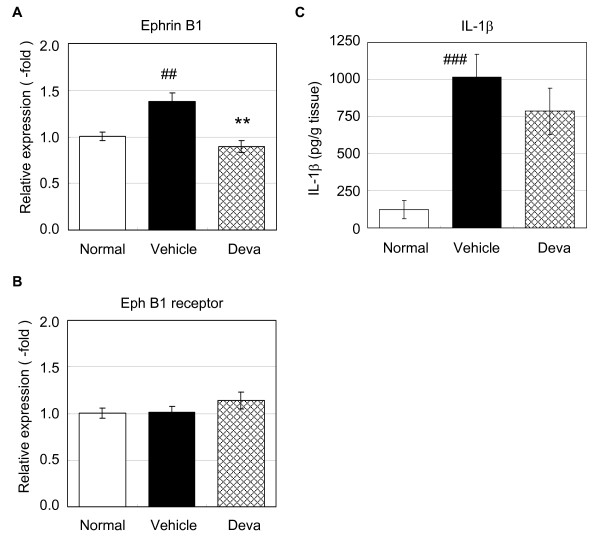
**Effects of devazepide on ephrin B1 and Eph B1 receptor gene expression and IL-1β production**. B16/BL6 cells were injected into the plantar region of the right hind paw of C57BL/6 mice, and devazepide (Deva, 10 mg/kg) was then administered orally once a day from day 7 after transplantation. A final oral administration of devazepide was given to mice prior to the collection of L4-5 DRG and right hind paw samples on day 14 after transplantation. Gene expression analyses for ephrin B1 (A) and the Eph B1 receptor (B) in DRGs were performed by quantitative real-time RT-PCR. Data represent fold changes versus the mean for normal mice. Columns represent the mean ± S.E. (n = 12). ##, p < 0.01, versus normal (t-test); **, p < 0.01, versus vehicle (t-test). C. Right hind paw samples were subjected to the extraction of IL-1β which was then measured by ELISA. The data are reported as the mean ± S.E. (n = 12). ###, p < 0.001, normal versus vehicle (Wilcoxon test); p = 0.08 (devazepide treatment), vehicle versus devazepide (Wilcoxon test).

In our previous study, repeated oral administration of devazepide, a CCK1 receptor antagonist, showed a similar anti-allodynic effect in the mouse model of cancer-induced pain to that of Z-360 [10]. Here, we examined the effects of devazepide on ephrin B1 gene expression in DRGs and the level of IL-1β in the cancer-inoculated region in the cancer-induced pain model in mice. Devazepide (10 mg/kg) was administered orally once a day from day 7 to 14 after transplantation, and gene expression of ephrin B1 and the Eph B1 receptor in L4-5 DRGs and the amount of IL-1β protein in the cancer-inoculated region were then measured. The repeated administration of devazepide significantly suppressed the expression of the ephrin B1 gene compared with the vehicle-treated group in cancer-induced pain mice (p < 0.01) (Figure 6A); however, the gene expression of the Eph B1 receptor was unchanged in all of the test groups (Figure 6B).

The amount of IL-1β protein in the cancer-inoculated region was reduced by 25.8% in the devazepide-treated group compared with vehicle-treated group (Figure 6C). The p-value of the Wilcoxon test compared with the vehicle-treated group was 0.08. The effect of devazepide on tumour size was assessed at day 14 after transplantation and no differences in the tumour size were detected between the control and devazepide-treated group (data not shown).

### Sulfated cholecystokinin-8 induced IL-1β production in the cancer-inoculated region

**Figure 7:**
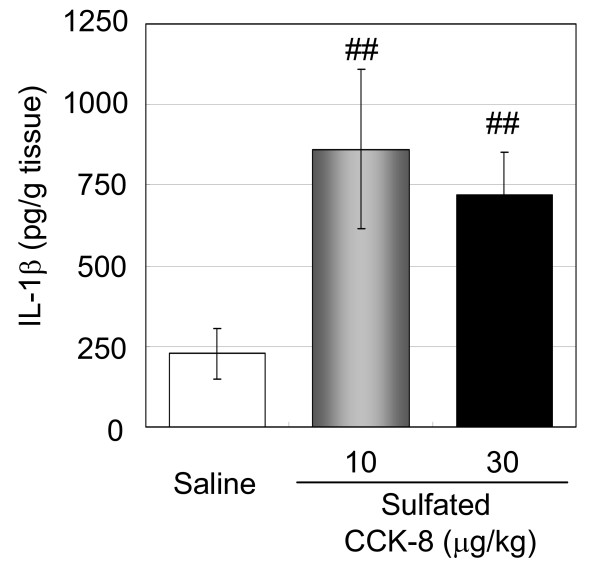
**Effect of sulfated CCK-8 on IL-1β production in the cancer-inoculated region**. B16/BL6 cells were injected into the plantar region of the right hind paw of C57BL/6 mice, and sulfated CCK-8 (10 or 30 μg/kg) was then administered by intraperitoneal injection twice a day from day 7 after transplantation. On day 10 after transplantation, a final dose of sulfated CCK-8 was administered to mice 30 min prior to the collection of right hind paw samples, which were the homogenized and subjected to ELISA for the measurement of IL-1β. The data are reported as the mean ± S.E. (n = 11-12). ##, p < 0.01, versus saline (non-parametric Dunnett's test).

To determine whether CCK contributed to the increased levels of IL-1β in the cancer-inoculated region, we examined the effect of sulfated CCK-8 injection on IL-1β production. Sulfated CCK-8 (10 and 30 μg/kg) was intraperitoneally injected into the mouse model of cancer-induced pain twice a day beginning from day 7 after transplantation, and the final injection was performed 30 min prior to the collection of paw samples on day 10. The levels of IL-1β protein in the cancer-inoculated regions were then measured by ELISA, which revealed that the administration of sulfated CCK-8 increased the amount of IL-1β protein compared with that of the vehicle-treated control group (p < 0.01) (Figure 7). The levels of IL-1β between the saline, 10 μg-, and 30 μg-sulfated CCK-8 treatment groups were 227.4, 862.0, and 719.1 pg/g tissue, respectively.

## Discussion

In this study, we discovered a novel pain cascade associated with the analgesic action of Z-360 in a mouse model of cancer-induced pain. In this cascade, IL-1β production from cancer-inoculated regions induces the gene expression of ephrin B1 in DRGs, which then ephrin B1 enhances the tyrosine phosphorylation of NR2B via Eph B receptor in the spinal cord, finally leading to pain. Z-360 was found to inhibit this pain cascade, suggesting that Z-360 reduces cancer-induced pain through the suppression of IL-1β production in cancer-inoculated regions, which in turn inhibits ephrin B1 gene expression, and tyrosine phosphorylation of NR2B. The pre-clinical results presented here indicate that Z-360 may provide relief against severe, opioid-resistant pain in patients with advanced pancreatic cancer.

In the cancer-induced pain model used in this study, it was reported that bradykinin and related peptides released from melanoma cells might cause spontaneous pain and allodynia [29]; however, we identified that the enhancement of IL-1β production, which may be predominantly released from immune cells invading tissues surrounding tumours, is another potential factor for causing pain. We demonstrated that IL-1β increased in cancer-inoculated regions and induced the expression of the ephrin B1 gene in the periphery of DRGs. We also found that the tyrosine phosphorylation of NR2B in the spinal cord was enhanced in the cancer-induced pain model in mice. The activation of the NMDA receptor is essential for the development of chronic inflammatory and neuropathic pain [30-32]. As our present data suggests that the novel pain cascade may be important for the development of cancer-induced pain, we propose that the components of this novel pain cascade, IL-1β (cancer-inoculated region) → ephrin B1 (DRGs) → NR2B (spinal cord), represent potential drug targets for the treatment of cancer-induced pain.

*In vitro *experiments have revealed that Z-360 has affinity for both recombinant human CCK2 and CCK1 receptors, with K_i_s of 0.47 and 316 nM, respectively [7]. In our previous study, the analgesic effect of CCK1 receptor antagonist, devazepide, was also observed after repeated administration to the mouse model of cancer-induced pain [10]. Here, devazepide (10 mg/kg) reduced the level of IL-1β protein in cancer-inoculated regions, and significantly suppressed the gene expression of ephrin B1 in DRGs to a similar degree as Z-360. Cunningham et al. reported that CCK induces the production of inflammatory cytokines, including IL-1β, in monocytes from peripheral blood [33]. We also confirmed here that the intraperitoneal injection of sulfated CCK-8 (10 and 30 μg/kg) resulted in an increase in IL-1β production in cancer-inoculated regions. Taken together, these results suggest that Z-360 is able to suppress IL-1β production via the blockade of CCK1 receptor.

In a previous pre-clinical study, Z-360 also exhibited analgesic effects on formalin-induced pain [10], and Honore et al. demonstrated that IL-1αβ gene-deficient mice display reduced nociceptive sensitivity to formalin-induced pain [23]. Although the detailed mechanisms are unclear, we speculate that Z-360 inhibits formalin-induced pain via the suppression of IL-1β production. It is also possible that other factors besides the suppression of IL-1β production participate in the analgesic effects of Z-360 in these pain models. The group of Grossi et al. reported that novel 1,5-benzodiazepine derivatives, such as Z-360, display analgesic and/or anti-inflammatory properties in spite of lacking affinities for central and peripheral benzodiazepine receptors [34,35]. A few of the examined derivatives showed an analgesic effect on acetic acid-induced writhing in mice, whereas other derivatives inhibited leukocyte migration via the suppression of nociceptive and/or inflammatory mediators, such as IL-6 and prostaglandin E2, in a carrageenan-induced air pouch model in mice. Furthermore, although the target molecules of these activities were not specified, it was found that a few derivatives had both analgesic and anti-inflammatory activities with low acute toxicity. These results indicate that Z-360 may be able to mediate analgesic action through other mechanisms in addition to the inhibition of IL-1β production.

Recently, a number of 1,5-benzodiazepine derivatives have been developed as drug candidates for diseases associated with pain [36,37], inflammation [38,39], nerve injury [40], and cardiovascular disorders [41]. Due to the related nature of their structures, the characterised chemical structure of Z-360 can provide important information for the design and development of innovative analgesics and anti-inflammatory agents.

In summary, we have identified a novel pain cascade, IL-1β (cancer-inoculated region) → ephrin B1 (DRGs) → NR2B (spinal cord) → pain, that may be responsible for the development of opioid-resistant pain in the mouse model of cancer-induced pain. Moreover, Z-360 inhibited the novel pain cascade through the suppression of IL-1β production. Accordingly, we consider that the suppression of IL-1β production by Z-360 was critical for its ability to alleviate cancer-induced pain in a previous clinical study [6].

## Conclusions

This pre-clinical study identified a novel pain cascade involving the release of IL-1β from a cancer-inoculated region, induction of ephrin B1 gene expression in DRGs, and subsequent enhancement of tyrosine phosphorylation of NR2B in the spinal cord. Notably, the oral administration of Z-360 inhibited the novel pain cascade through the suppression of IL-1β production. We conclude that the pre-clinical results presented here support the relief action of Z-360 against severe, opioid-resistant pain in patients with advanced pancreatic cancer. Pre-clinical and clinical results have demonstrated that Z-360 combined with gemcitabine is a safe and promising pancreatic cancer drug with characteristic analgesic effects in addition to the prolongation of survival.

## Methods

### Animals

Five and seven-week-old male C57BL/6NCrlCrlj (C57BL/6) mice were purchased from Charles River Laboratories Japan, Inc. (Yokohama, Japan). Mice were kept under controlled temperature (23 ± 3°C), humidity (55 ± 20%), and lighting (lights on at 7:00 AM and of at 7:00 PM) conditions, and food and water were available ad libitum. All experimental procedures were approved by the Institutional Animal Care and Use Committee of Zeria Pharmaceuticals Central Research Laboratories. Samplings were carried out between 10:00 AM and 4:00 PM.

### Drug

Z-360 was synthesized at the Central Research Laboratories of Zeria Pharmaceutical Co., Ltd. (Kumagaya, Japan). Devazepide was purchased from Tocris Bioscience Inc. (St. Louis, MO, USA). Z-360 and devazepide were resuspended in a 0.5% (w/v) carboxymethyl cellulose sodium solution before use.

### Mouse model of cancer-induced pain

A mouse model of cancer-induced pain was made according to the method of Sasamura et al [11]. Briefly, B16/BL6 melanoma cells derived from C57BL/6 mice (Cell Resource Center for Biomedical Research, Tohoku University, Sendai, Japan) were cultured in RPMI1640 medium containing 10% fetal bovine serum at 37°C and a humidified atmosphere of 5% CO_2_. Cells (2 × 10^5 ^cells/20 μL) were injected into the plantar region of the right hind paw of six-week old C57BL/6 mice. Z-360 (100 mg/10 mL/kg) or devazepide (10 mg/10 mL/kg) was administered orally once a day from day 7 to 14 after transplantation, and paw withdrawal thresholds were measured on day 7 and 14. Z-360 or devazepide (at the above-mentioned concentrations) was administered orally 60 min prior to tissue sampling on day 14 after transplantation.

### Preparation of DRG samples for gene expression analysis

On day 14 after transplantation, L4 and L5 DRG samples were taken from normal mice and the cancer-induced pain mouse model. Two DRG bodies for each sample were pooled and then homogenized with lysis buffer from an RNeasy Micro Kit (QIAGEN, Hilden, Germany) using a Tissue Lyser (QIAGEN) for the preparation of total RNA.

### Real-time quantitative RT-PCR

Total RNA was prepared from tissue lysate using an RNeasy Micro Kit (QIAGEN). After purification, the amount of RNA was measured spectrophotometrically using OD260, and the quality of RNA was checked by gel electrophoresis and spectrophotometric analysis using OD260/280. Total RNA (100 ng) was converted into first-strand cDNA by PrimeScript RT Reagent Kit (Takara Bio Inc., Otsu, Japan). The resulting first-strand cDNAs were subjected to real-time quantitative RT-PCR with ABsolute™ QPCR ROX Mix (Thermofisher Scientific, Waltham, MA, U.S.A.) and TaqMan probes for ephrin B1 (Mm00438666_m1) and the Eph B1 receptor (Mm00557961_m1), using an ABI PRISM 7900HT Sequence Detection System (Applied Biosystems, Foster City, CA, U.S.A.). The cycling parameters were 15 min at 95°C followed by 45 cycles of 15 sec at 95°C and 1 min at 60°C. The obtained data were normalized to the expression of GAPDH (Mm99999915_g1) and analyzed by the Comparative CT Method (Applied Biosystems).

### Tyrosine phosphorylation of NR2B

The relative tyrosine phosphorylation of NR2B was examined by immunoprecipitation. For the analysis, the spinal cords from normal and cancer-induced pain model mice were rapidly removed on day 14 after transplantation. Z-360 (100 mg/kg) was administered orally once a day beginning from day 7 after transplantation. Tissues were homogenized in solubilization buffer containing 50 mM Tris-HCl (pH 8.0), 150 mM NaCl, 1 mM EDTA (pH 8.0), 1% NP-40, 0.1% sodium dodecyl sulfate (SDS), 0.5% sodium deoxycholate, 1 mM sodium vanadate (Na_3_VO_4_), 25 mM sodium fluoride (NaF), 1 mM PMSF, and protease inhibitor cocktail (Sigma-Aldrich, St Louis, MO, U.S.A.) using a Tissue Lyser (QIAGEN). After centrifugation at 12,000 rpm for 5 min at 4°C, the supernatants containing the proteins were collected and the protein concentrations were determined using the BCA Protein Assay (Bio-Rad Laboratories, Hercules, CA, U.S.A.). Equal amounts of proteins were added to 0.6 μg of rabbit anti-NR2B antibody (Millipore, Billerica, MA, U.S.A.) and gently shaken overnight at 4°C. The following morning, Dynabeads Protein G (Invitrogen, Carlsbad, CA, U.S.A.) were added to the samples which were then gently shaken for 1 h at 4°C. Beads were then rinsed in solubilization buffer and boiled in loading buffer (2% SDS, 50 mM Tris-HCl (pH 6.8), 200 mM β-mercaptoethanol, 10% glycerol, and 0.1% Bromophenol Blue) for 5 min. The protein-rich supernatants were separated on a 7.5% Ready-gel J (Bio-Rad Laboratories) and transferred to a polyvinylidine difluoride (PVDF) membrane using a Trans-Blot Cell System (Bio-Rad Laboratories). The membranes were blocked for 1 h at room temperature with 3% non-fat milk in Tris buffered saline containing 20 mM Tris-HCl (pH 7.5), 150 mM NaCl, and 0.1% Tween20 (TBST) and then incubated overnight at 4°C with rabbit anti-phospho-tyr1472-NR2B antibody (1:1000, Upstate, Cambridge, U.K.). After washing with TBST, membranes were incubated for 1 h at room temperature with HRP-rec-Protein G (1:8000, Invitrogen). All antibodies were diluted in TBST. Protein bands were visualized using an Enhanced Chemi-Luminescence Detection Kit (ECL Plus, Amersham Pharmacia, Chalfont St.Giles, U.K.) followed by autoradiography using Hyperfilm (Amersham Pharmacia). The blots were then washed in TBST and stripped for 30 min in stripping buffer containing 2% SDS, 62.5 mM Tris-HCl (pH 6.8), and 100 mM β-mercaptoethanol. The membranes were washed twice in TBST for 30 min and then blocked for 1 h at room temperature with 3% non-fat milk in TBST. Membranes were reprobed for total NR2B by overnight incubation at 4°C with rabbit anti-NR2B antibody (Millipore). Blots were then washed, incubated for 1 h at room temperature with HRP-rec-Protein G (1:8000, Invitrogen), and visualized as above. Hyperfilms were scanned and captured using Adobe Photoshop Element 5.0 software (Adobe Systema Inc., Scion Jose, CA, U.S.A.) for densitometric quantification. We obtained the relative phosphotyrosin-1472-NR2B band density (relative pTyr1472-NR2B density) levels by normalizing the anti-phosphotyrosine immunoblot against the corresponding total NR2B immuoblot from identical samples.

### IL-1β levels in the right hind paw

On day 14 after transplantation, we dissected the cancer-inoculated regions of right hind paw including B16/BL6 melanoma or normal as sample. After measuring their weight, samples were homogenized in 0.5 mL protein lysis buffer containing 10 mM Tris-HCl (pH 7.5), 150 mM NaCl, 1 mM EDTA (pH 8.0), 1% NP-40, 1 mM sodium vanadate (Na_3_VO_4_), 1 mM PMSF, and protease inhibitor cocktail (Sigma-Aldrich) using an Ultra-Turrax T25 (Janke & Kunkel, IKA Werk, Staufen, Germany). After centrifugation at 3,000 rpm for 15 min at 4°C, supernatants containing the proteins were collected and IL-1β levels were determination by ELISA (R&D Systems, Minneapolis, MN, U.S.A.).

### IL-1β-inducible gene expression analysis in DRGs

Recombinant mouse IL-1β (R&D systems) (5 μL, 300 or 1000 pg/paw) was injected into the plantar region of the right hind paw of eight-week old C57BL/6 mice. Sixty min after the injection, L4-5 DRG samples were removed and gene expression analysis and real-time quantitative RT-PCR were performed as described above.

### Sulfated CCK-8-induced IL-1β production

In the mouse model of cancer-induced pain, intraperitoneal injection of 10 mL of sulfated CCK-8 per kg body weight (10 or 30 μg/kg, Peptide Institute, Inc., Osaka, Japan) was performed twice daily from day 7 after transplantation. On day 10, the amounts of IL-1β protein in the right hind paw samples were measured by ELISA as described above. Sulfated CCK-8 was administered 30 min prior to the removal of the paw. In total, the injection of sulfated CCK-8 was performed seven times.

### Statistical analysis

All data are expressed as the mean ± standard error (S.E.). Data were analyzed with SAS System Version 8.2 (SAS Institute Japan Ltd., Tokyo, Japan) using the t-test, Wilcoxon signed-rank test, and non-parametric Dunnett's test. Differences with a p-value of less than 0.05 were considered statistically significant.

## Competing interests

The authors declare that they have no competing interests.

## Authors' contributions

YO participated in the experimental design, carried out the experiments and data analysis, and drafted the manuscript. KS participated in the experimental design, helped perform the experiments, provided data interpretation, and finalized the manuscript. NK, HH, and YH helped perform the experiments. HK, KY, TM, and MT finalized the manuscript. All authors read and approved the final manuscript.
